# Living donor liver transplantation for intra hepatic cholangiocarcinoma

**DOI:** 10.1016/j.amsu.2020.07.028

**Published:** 2020-07-21

**Authors:** Abu Bakar Hafeez Bhatti, Rizmi Tahir, Najla Rahman Qureshi, Nadira Mamoon, Nusrat Yar Khan, Haseeb Haider Zia

**Affiliations:** a Department of HPB Surgery and Liver Transplantation, Shifa International Hospital, Islamabad, Pakistan; b Department of Pathology, Shifa International Hospital, Islamabad, Pakistan

**Keywords:** Cholangiocarcinoma, Liver transplant, Survival

Living transplantation is an established treatment for patients with hepatocellular carcinoma (HCC) and cirrhosis [1]. Liver transplantation for intra hepatic cholangiocarcinoma (i-CC) or mixed hepatocellular-cholangiocarcinoma(h-CC) is not performed routinely. In most centers, i-CC is considered a contraindication to liver transplantation. There is limited published literature with regards to outcomes of LT in i-CC or h-CC and remain inferior when compared with HCC. Results are mainly derived from incidental finding of CC on explant histopathology in patients transplanted for suspected HCC, predominantly in deceased donor liver transplant (DDLT) setting [2]. Living donor liver transplantation (LDLT) is an acceptable alternative to DDLT for HCC. The largest report for i-CC/h-CC included 11 patients and reported a 3 year recurrence free survival (RFS) of 46.7% [3].

In our center, LDLT is not offered to patients with a diagnosis of i-CC or h-CC preoperatively. As a busy LDLT center, HCC contributes to a major portion of our transplant activity, but occasionally, these patients are found to have i-CC or h-CC on explant histopathology. Here, we share our experience with a relatively large cohort of patients who underwent LDLT for incidental i-CC/h-CC.

Between April 2012 and December 2019, 898 LDLTs were performed and 16 patients were found to have i-CC or h-CC on explant histopathology and were included. Diagnosis of HCC was confirmed on a liver dynamic CT scan and/or MRI scan. Biopsy was reserved for cases where diagnosis could not be established on imaging. Extrahepatic metastasis and main portal vein tumor invasion were considered absolute contraindications to transplantation. Down staging protocol for larger tumors and use of pre transplant treatments as a bridge to transplantation have been previously detailed [4]. After transplantation, patients diagnosed with i-CC or h-CC were kept under surveillance with 3–6 monthly CT scan and AFP and CA-19-9 for 6 months, then for 2 years and annually thereafter.

For the purpose of this study, etiology, MELD scores, and histopathological variables were assessed. Site of recurrence and number of involved organs were also determined. Recurrence free survival (RFS) was determined by subtracting date of recurrence from date of transplantation. Kaplan Meier curves were used for survival analysis and Breslow's test was used to determine significance. For assessment of recurrence and RFS, patients who expired within 3 months post transplant were excluded. A P value < 0.05 was considered statistically significant. Analysis was performed on SPSS version 22. The hospital ethics committee approved the study.

Median age was 52(40-68) years. Male to female ratio was 15:1. Hepatitis C infection was the underlying etiology in 15/16(93.7%) and HBV-HDV in 1/16(6.3%) patients. Median AFP was 65.8(5.6–2048)ng/ml. Median MELD score was 18(12-30). Out of 7 patients who received pre transplant treatment, 6 underwent transarterial chemoembolization and (TACE) and 3 had pre transplant radiofrequency ablation (RFA). The overall mortality was 9/16(56%). Out of these, 3 patients died within 3 months due to sepsis. Out of 13 patients, 6(46.1%) had recurrence in the follow up period. The most frequent site of recurrence was bone and transplanted graft in 4/6(66.7%) patients each. The overall 3 year RFS was 47% (Fig. 1). Out of all the histopathological variables, a significant difference in estimated 3 year RFS was seen in patients with poor grade (64% vs 22%, P = 0.03) as shown in Table 1.

**Fig. 1:**
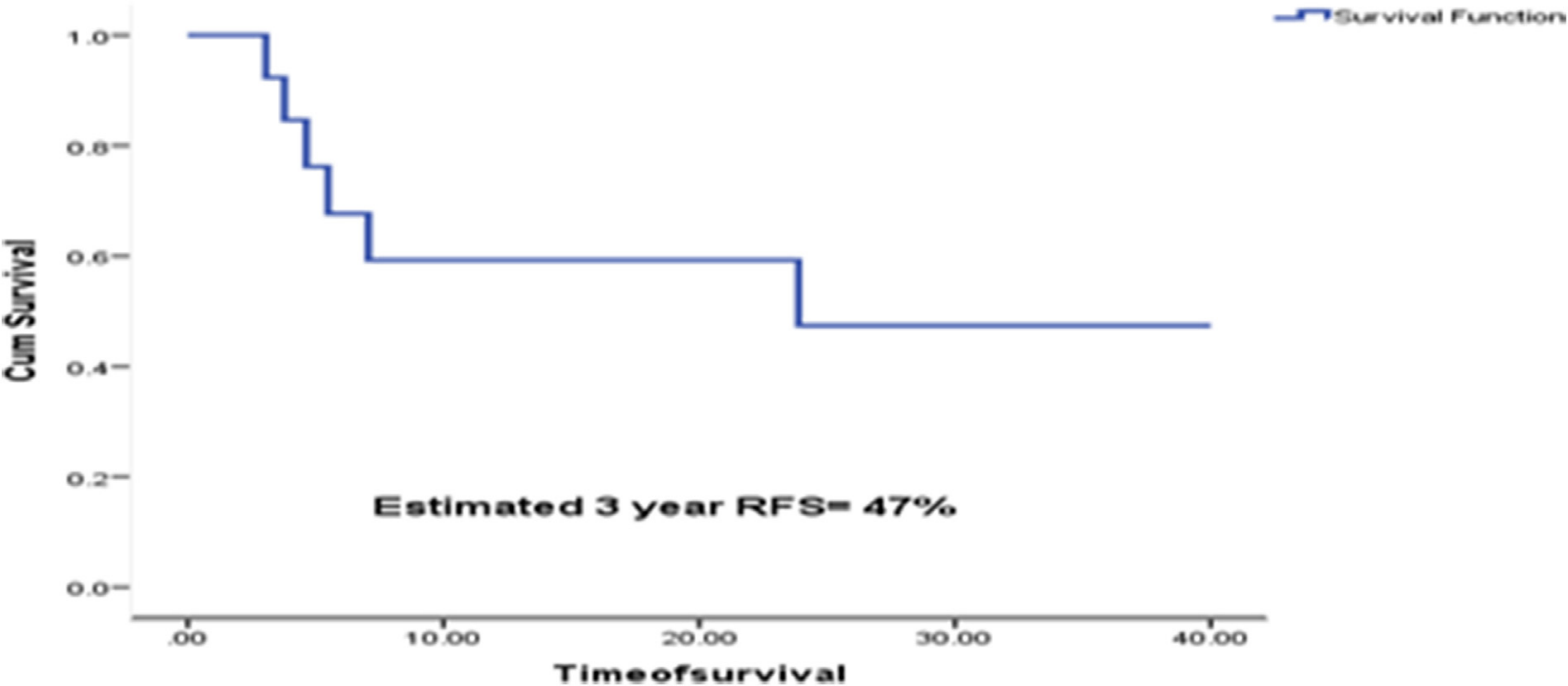
Estimated 3 year recurrence free survival after living donor liver transplantation for intra hepatic cholangiocarcinoma.

**Table 1:** Histopathological variables and recurrence free survival.

		Number	Percent	3 year RFS (n = 13)	P value
Tumor size	<5 cm	9	56.2	58	0.1
	≥5 cm	7	43.8	31	
Grade	Well-moderate	9	56.2	64	0.03
	Poor	7	43.8	22	
Microvascular invasion	Present	8	50	67	0.5
	Absent	8	50	34	
Final diagnosis	Intra hepatic cholangiocarcinoma	9	56.2	63	0.1
	Mixed hepatocellular-cholangiocarcinoma	7	43.8	33	

The current study reports one of the largest single center experience with LDLT for i-CC and mixed h-CC. Although the overall RFS was unacceptable, a subgroup of patients with well-moderately differentiated tumors might have promising outcomes. Based on inferior outcomes after LT for i-CC/h-CC, when compared with HCC, and potential risk to a living donor, resection appears to be a better option for patients when a preoperative diagnosis is established. There was no significant difference in survival when patients with i-CC/h-CC underwent surgical resection versus transplant. However, when patients have accompanying liver failure, resection is not an option and role of liver transplantation remains unclear [5].

There is great interest in identifying a group of patients with i-CC/h-CC who can potentially benefit from transplantation and demonstrate long term survival. Very small tumors and favorable biology has been shown to yield outcomes comparable to HCC [2]. In a matched study on early CC(<2 cm, single lesion) and HCC, the one and five year survival was 63.6% and 90% and 63.6% and 70.3%(P = 0.25) [6].

In the current study, we an observed a significant difference in survival between patients based on tumor grade. The 3 year RFS was 64% in patients with well-moderately differentiated i-CC/h-CC. These findings demonstrate a promising role of LT, in patients with liver failure and early CC with favorable tumor biology. The potential group of patients who would really benefit from LT needs to carefully identified, perhaps in the setting of a trial. The main limitation of the current study was the small sample size which limits the clinical applicability of the results. This is mainly because LT is not performed for CC outside experimental protocols and remains an incidental finding on explant histopathology. Nevertheless this is still the largest report assessing the role of LDLT in these patients.

For liver transplantation, i-CC/h-CC should be considered a relative contra indication. Patients with favorable biology and underlying liver failure may occasionally be considered transplant candidates. Further research is required to identify the most suitable group that will benefit from LT and achieve acceptable long term survival.

## Ethical approval

Exemption from formal review was given by hospital ethics committee.

## Sources of funding

No funding was received.

## Author contribution

AHB: Concept, design, analysis, interpretation, drafting the work and final approval.

RT and NRQ: Data collection and analysis, drafting the manuscript, final approval.

NM, NYK, HHZ: Drafting the manuscript, final approval.

## Trial registry number

This is a short communication for the general interest of the transplant community and was not registered.

## Guarantor

Abu Bakar Hafeez Bhatti.

## Provenance and peer review

Not commissioned, externally peer reviewed.

## Declaration of competing interest

There are no conflicts of interests.
